# Increased miR-6132 promotes deep vein thrombosis formation by downregulating FOXP3 expression

**DOI:** 10.3389/fcvm.2024.1356286

**Published:** 2024-03-20

**Authors:** Yunhong Zhang, Zhen Zhang, Haoyang Li, Chu Chu, Gang Liang, Nannan Fan, Ran Wei, Tingting Zhang, Lihua Li, Bin Wang, Xia Li

**Affiliations:** ^1^ Innovative Institute of Chinese Medicine and Pharmacy, Shandong University of Traditional Chinese Medicine, Jinan, Shandong Province, China; ^2^ International Business School, Tianjin Foreign Studies University, Tianjin, China; ^3^ Department of Peripheral Vascular Disease, Affiliated Hospital of Shandong University of Traditional Chinese Medicine, Jinan, Shandong Province, China; ^4^ School of Clinical and Basic Medical Sciences, Shandong First Medical University& Shandong Academy of Medical Sciences, Jinan, Shandong Province, China

**Keywords:** deep vein thrombosis, FOXP3, miR-6132, immune response, post-transcriptional regulation

## Abstract

**Background:**

Deep vein thrombosis (DVT) is associated with aberrant gene expression that is a common peripheral vascular disease. Here, we aimed to elucidate that the epigenetic modification of forkhead box protein 3 (FOXP3) at the post-transcriptional level, which might be the key trigger leading to the down-regulation of FOXP3 expression in DVT.

**Methods:**

In order to explore the relationship between microRNAs (miRNAs) and FOXP3, mRNA and microRNA microarray analysis were performed. Dual luciferase reporter assay was used to verify the upstream miRNAs of FOXP3. Quantitative real-time polymerase chain reaction, flow cytometry and Western blot were used to detect the relative expression of miR-6132 and FOXP3. Additionally, DVT models were established to investigate the role of miR-6132 by Murine Doppler Ultrasound and Hematoxylin-Eosin staining.

**Results:**

Microarray and flow cytometry results showed that the FOXP3 expression was decreased while miR-6132 level was increased substantially in DVT, and there was significant negative correlation between miR-6132 and FOXP3. Moreover, we discovered that overexpressed miR-6132 reduced FOXP3 expression and aggravated DVT formation, while miR-6132 knockdown increased FOXP3 expression and alleviated DVT formation. Dual luciferase reporter assay validated the direct binding of miR-6132 to FOXP3.

**Conclusion:**

Collectively, our data elucidate a new avenue through which up-regulated miR-6132 contributes to the formation and progression of DVT by inhibiting FOXP3 expression.

## Introduction

Deep vein thrombosis (DVT) is the formation or presence of a thrombus in the deep veins, that most commonly occurs in the large veins of the legs or pelvis. Pulmonary embolism (PE) is a terrible complication of DVT and the primary contributor to mortality, PE and DVT are known as venous thromboembolism (VTE) (1, 2). The annual incidence of DVT is about 1 per 1,000 population and it seriously affects the health and quality of patients' life (3, 4). Incidence is steadily rising due to association with many types of risk factors, such as population ageing, genetics, obesity, lifestyle, malignancy, autoimmune diseases, and infection (5, 6). Nevertheless, autoimmune diseases are frequently associated with venous thrombotic events (7). In order to find new targets for the treatment of DVT, it deeply desiderates to reveal the underlying molecular mechanism of DVT formation.

In previous studies, it was found that the proportion of Regulatory T cells (Tregs) subgroups in DVT model rats were significantly reduced (8). Tregs are a subset of T-cells that inhibit immune responses and play an important role in maintaining immune tolerance. Meanwhile, Tregs also can negatively regulate varieties of physiological and pathological immune responses (9). As well known, Tregs express surface molecules of CD4 and CD25, secret anti-inflammatory cytokines represented by IL-10 or TGF-β1 (10, 11). However, the most important transcription factor for Tregs is Forkhead box protein 3 (FOXP3), which plays an important role in their development and differentiation (12). FOXP3 is a 48 kD protein composed of 431 amino acids and is also a member of the fork head transcription factor family (13, 14). The human gene of FOXP3 consists of 11 exons and exists in the p arm of the X chromosome (Xp11.23), while the mouse gene at X A1.1 is equivalent to that of the human gene (15, 16). It has also been confirmed that FOXP3 signaling pathways regulate the process of immune, but the role of FOXP3 and its upstream regulatory factors remain ambiguous in the formation of DVT.

MicroRNAs (miRNAs) are small endogenous noncoding RNAs (typically 21–23 nucleotides in length) that negatively regulate gene expression via recognition of cognate sequences and interference of transcriptional, translational or epigenetic processes (17–19). As posttranscriptional regulators of gene expression, miRNAs have huge potential biological functions in the physiological and pathological processes of various diseases (20, 21). Interestingly, dysregulation of miRNAs expression was shown to be associated with various of cardiovascular diseases, such as atherosclerosis and DVT (22, 23). As a result, we speculated that certain miRNAs acting on FOXP3 might represent novel targets for DVT treatment.

In this study, we observed that FOXP3 expression was decreased and miR-6132 was increased in DVT patients. Notably, miR-6132 bound to its 3′UTR and thus effectively suppressed FOXP3 expression. Meanwhile, we found that increasing miR-6132 could aggravate DVT formation by inhibiting FOXP3 expression, while inhibiting miR-6132 could alleviate the formation of DVT by enhancing FOXP3 expression. Therefore, the newly discovered miR-6132/FOXP3 axis might offer new strategies for DVT treatment.

## Methods and materials

### Patients

A total of 36 patients diagnosed with symptomatic first idiopathic DVT of the lower limbs, and 36 control subjects in the Affiliated Hospital of Shandong University of Traditional Chinese Medicine from July 2018 to July 2022 were enrolled in this study. The involved DVT patients were made a definite diagnosis by Color Doppler Ultrasound and lower extremity angiography without a history of diabetes mellitus, hypertension or other chronic diseases (Table 1). This study was written informed consent was obtained from all participants and approved by the Ethics Committee of Shandong University of Traditional Chinese Medicine.

**Table 1:** Baseline characteristics of DVT patients and healthy controls.

Characteristics	Control (*n* = 36)	DVT (*n* = 36)
Age, years (mean ± SD)	54.1 ± 8.7	57.3 ± 9.9
Gender, females/males	17/19	16/20
Recent immobilization/surgery	0	0
Anti-coagulants or platelet-inhibitors	0	0
Hormone	0	0
Smoking	0	0
Hypertension	0	0
Diabetes mellitus	0	0
Other chronic diseases	0	0

### Specimen collection

Venous blood samples were drawn in the fasting state for approximately 12 h and the samples were encoded for blind analysis. Peripheral blood mononuclear cells (PBMCs) were separated by Ficoll density-gradient centrifugation.

### Microarray analysis

The genome-wide analysis of mRNA and miRNA expression in DVT patients (*n* = 6) and control subjects (*n* = 6) were performed using Human mRNA and miRNA (4 × 180 K, Design ID: 084410, 8 × 60 K, Design ID: 070156). Sample labeling, microarray hybridization and washing were performed by OE Biotech. Co., Ltd. (Shanghai, China) according to the manufacturer's standard protocols.

### Cell culture and transfection

293T cells were obtained from Procell Life Science & Technology Co., Ltd (Wuhan, China). 293T cells were transfected with miR-6132 mimics, inhibitor or respective negative control (GenePharma, Shanghai, China). The transfected cells were cultured in RPMI 1640 culture medium and harvested for 24 h. The sequences used in this study are listed in Table 2.

**Table 2:** The sequences of synthesized mimics, negative control (NC), inhibitor and inhibitor NC.

Gene	Sequences (5′-3′)
hsa-miR-6132 mimics	Sense: AGCAGGGCUGGGGAUUGCA
	Antisense: CAAUCCCCAGCCCUGCUUU
NC	Sense: UUCUCCGAACGUGUCACGUTT
	Antisense: ACGUGACACGUUCGGAGAATT
hsa-miR-6132 inhibitor	UGCAAUCCCCAGCCCUGCU
Inhibitor NC	CAGUACUUUUGUGUAGUACAA

### Quantitative real-time PCR (qRT-PCR)

Total RNA was extracted using TRIzol Reagent (Invitrogen, Carlsbad, USA) following specification. Approximately 1 µg of RNA was reverse transcribed into cDNA, which was using SYBR Green (Invitrogen, Carlsbad, USA) for qRT-PCR analysis, and the relative mRNA levels were calculated using the 2^−^*^ΔΔ^*^Ct^ formula. The miRNA primers are designed and synthesized by RiboBio (Guangzhou, China). The PCR primer sequences are shown in Table 3.

**Table 3:** Quantitative real-time PCR (qRT-PCR) primers used in this study.

Gene	Sequences (5'-3’)
GAPDH (homo sapiens)	F: GCACCGTCAAGGCTGAGAAC
	R: TGGTGAAGACGCCAGTGGA
Actin (mus musculus)	F: GGCTGTATTCCCCTCCATCG
	R: CCAGTTGGTAACAATGCCATGT
FOXP3 (homo sapiens)	F: CACTGCCCCTAGTCATGGTG
	R: CGTTGAGAGCTGGTGCATGA
Foxp3 (mus musculus)	F: ACTCGCATGTTCGCCTACTT
	R: TTGGCTCCTCTTCTTGCGAA

### Dual-luciferase reporter assay

The FOXP3 wild-type (WT) and FOXP3 mutant (MUT) mRNA 3′UTR luciferase reporter vectors were constructed severally. 293T cells were co-transfected with WT or MUT luciferase reporter plasmids and the miR-6132 mimics or negative controls at an ultimate concentration of 100 nM. Luciferase activity was measured in cell lysates using a dual-luciferase reporter system 24 h after transfection.

### Flow cytometric analysis

Cells were resuspended in 1×PBS and stained with the following conjugated antibodies: CD4, FOXP3 (eBioscience, Ben Lomond, CA, USA) or their corresponding isotypic control for 30 min at 4°C in the dark. It was detected using the flow cytometer (Beckman Coulter, CytoFLEX, California, USA). All data were analyzed by using FlowJo _v10.8.1 (Treestar, USA).

### Western blotting

Samples were lysed in RIPA buffer with protease and phosphatase inhibitor (Solarbio, Beijing, China), and the proteins were subjected to SDS-PAGE and transferred onto nitrocellulose membranes. Then, the membranes were incubated with diluted primary antibodies followed by horseradish peroxidase HRP-conjugated secondary antibody. Antibodies against FOXP3 (1:1,000, ab215206, Abcam, Cambridge, USA) and GAPDH (1:10,000, ab181603, Abcam, Cambridge, USA) were used in this study.

### DVT mice model and treatment

C57BL/6J mice (8-week-old male) were purchased from Beijing HFK Bioscience Company (Beijing, China). Animal experiments were approved by the Institutional Animal Care and Use Committee of Shandong University of Traditional Chinese Medicine. The model of DVT mice was constructed according to the inferior vena cava (IVC) stenosis method, as described above (24).

The mice were stochastically falled into 4 groups (15/group): (1) DVT NC group; (2) DVT miR-6132 mimics group; (3) DVT INC group; (4) DVT miR-6132 inhibitor group.

For FOXP3 inhibitor experiments, mice were stochastically split into 3 groups (15/group): (1) DVT + INC group; (2) DVT miR-6132 inhibitor group; (3) DVT miR-6132 inhibitor + Epirubicin (FOXP3 inhibitor, MCE, USA) group. NC (5 nmol), miR-6132 mimics (5 nmol), INC (10 nmol), or miR-6132 inhibitor (10 nmol) were directly injected into the tail vein before undergoing the surgery, respectively. Epirubicin (0.3 μg/g) was injected into the tail vein before the mice underwent IVC stenosis and continued for three consecutive days (25). The fresh specimen section 2 mm below the IVC ligation site were sectioned for Hematoxylin and eosin (H&E) analysis.

### Murine doppler ultrasound

Mice were anesthetized using a mixture of isoflurane-oxygen for Vascular Doppler ultrasonography. Thrombus formation images were obtained using Small Animal Ultrasound Imaging System (VINNO6 LAB, VINNO, Suzhou, China).

### Enzyme-linked immunoassay (ELISA)

The protein expressions of endothelial nitric oxide synthase (eNOS) and endothelin-1 (ET-1) were detected by ELISA assay following the manufacturer's instructions with mice ELISA Kits, following the manufacturer's instructions. The absorbance of 450 and 630 nm was measured using a microplate reader.

### H&E

After embedding in paraffin, the tissues were cut into 4 µm-thick sections and stained with H&E following standard procedures. All histological images were observed via light microscopy (E100, Nikon, Japan).

### Statistical analyses

The results are expressed as the mean ± standard error, and the 2-tailed Student's *t* test or one-way ANOVA were used for comparisons. Statistical analysis was performed with SPSS 16.0 and GraphPad Prism 8.0 software. The diagnostic value was evaluated by receiver operating characteristic (ROC) curve and correlations were analyzed by Pearson correlation. All experiments were representative of at least three times independently, unless otherwise noted. The values of *P *< 0.05 were considered statistically significant.

## Results

### FOXP3 was decreased under the condition of DVT

**Figure 1:**
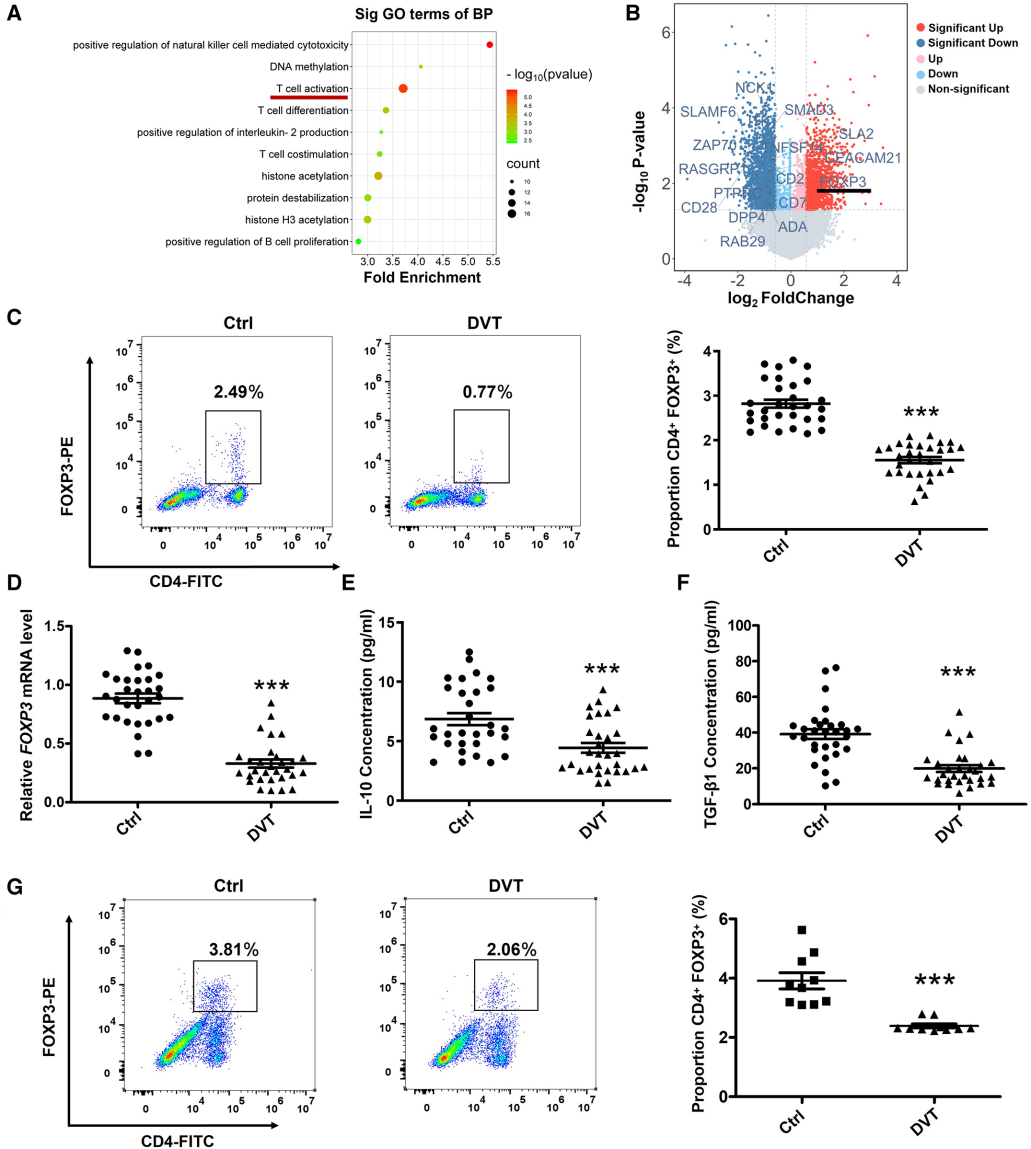
The expression of FOXP3, IL-10 and TGF-β1 are downregulated in DVT patients. (**A**) The top 10 gene ontology terms of biological process pathway enrichment analyses according to mRNA microarray analysis. (**B**) Volcano plot showing the profiling data of down-regulated mRNAs in the blood of six DVT patients compared with that of six controls determined by microarray analysis. Red indicates increased relative expression, while blue indicates decreased relative expression. (**C**,**D**) Relative expression levels of FOXP3 protein and mRNA in the peripheral blood mononuclear cells (PBMCs) from 30 DVT patients and 30 controls were determined by flow cytometry and quantitative real-time PCR (qRT-PCR). (**E**,**F**) IL-10 and TGF-β1 protein levels were determined by ELISA in plasma (*n* = 30). (**G**) FOXP3 protein levels were determined by flow cytometry in mice (*n* = 10). ***P < 0.001.

In order to demonstrate the physiological function and the pathogenesis of DVT, we performed an analysis of the biological processes involved in the downregulation of 1,866 differentially expressed genes (*P *< 0.05, FC > 1.5) using Gene Ontology (GO) terms. Among the top ten significantly enriched GO terms, we found differential enrichment of pathways involved in T cell activation (Figure 1A). Interestingly, we found FOXP3 was down-regulated in *T* cell activation pathway (Figure 1B). To identify the role of FOXP3 in the progression of DVT, we first examined the expression of FOXP3 and found that FOXP3 is relatively low expressed in DVT patients and DVT mice (Figures 1C,D,G). Meanwhile, the expression levels of IL-10 and TGF-β1 were also significantly reduced in DVT patients (Figures 1E,F).

### FOXP3 negatively correlated to miR-6132 in DVT patients

**Figure 2:**
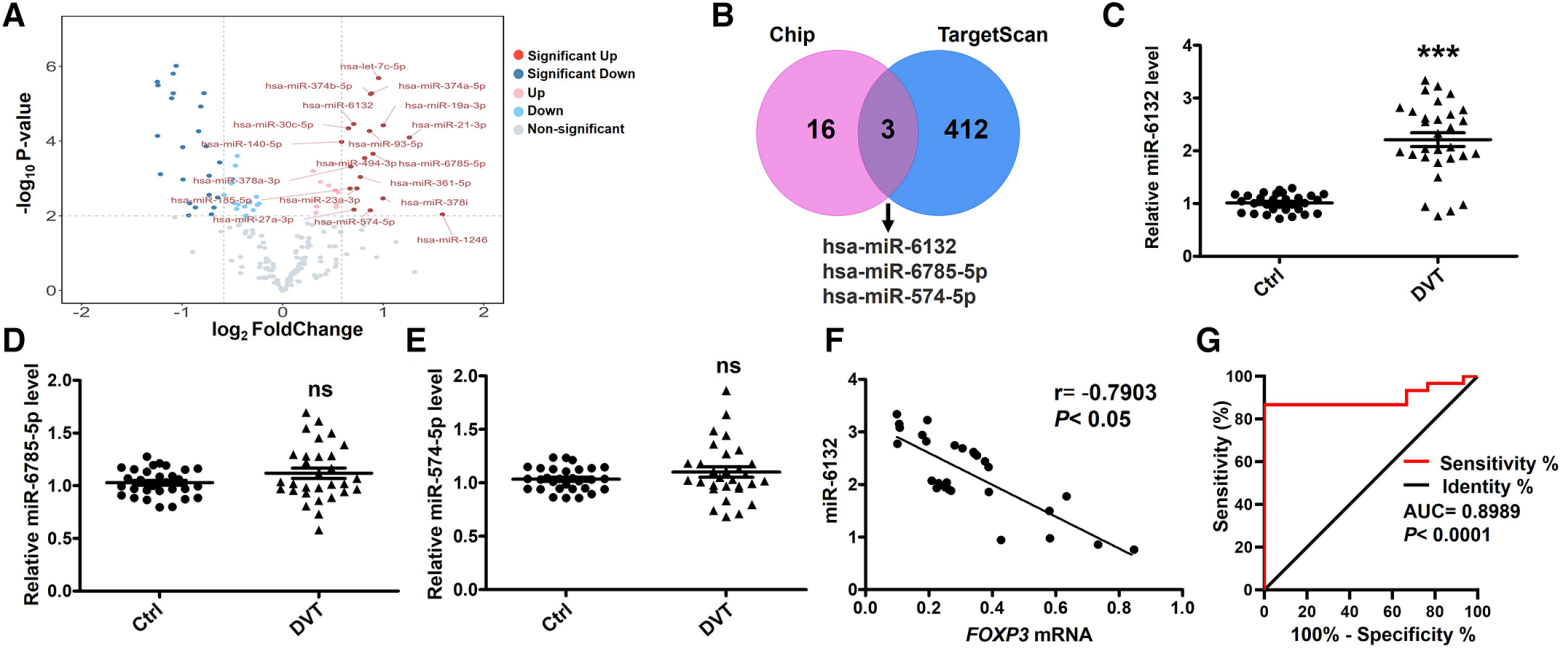
Negative correlation between the levels of miR-6132 and FOXP3 in patients with DVT. (**A**) Volcano plot showing the profiling data of up-regulated miRNAs in the blood of six DVT patients compared with that of six controls determined by microarray analysis. Red indicates increased relative expression, while blue indicates decreased relative expression. (**B**) The Venn diagram shows that miR-6132, miR-6785-5p and miR-574-5p were predicted by Targetscan and Chip. (**C**) Levels of miR-6132, miR-6785-5p and miR-574-5p in PBMCs from DVT patients (*n* = 30) compared with control subjects (*n* = 30) were measured by qRT-PCR. (**D**) The correlation between miR-6132 and FOXP3 was analyzed using Pearson correlation analysis (*n* = 30). (**I**) Diagnostic value of miR-6132 for DVT was evaluated by ROC curve. ***P < 0.001.

To further confirm the role of FOXP3 and miRNAs in DVT, we performed miRNA expression profiling. The mostly up-regulated 19 miRNAs were detected by at least 1.5-fold with *P *< 0.01 in DVT (Figure 2A). Here, we intersected the data from the TargetScan database and Chip, and displayed the possible interaction between miR-6132, miR-6785-5p, miR-574-5p, and FOXP3 (Figure 2B). Using amplified samples for validation, miR-6132 was robustly increased in DVT (Figures 2C–E). Next, miR-6132 was negatively correlated with FOXP3 (Figure 2F). With an area under the curve of 0.8989 (95% confidence interval: 0.8036–0.9942), ROC analysis indicated that miR-6132 was sensitive to distinguish DVT patients from healthy control subjects (Figure 2G). Together, our results suggested that miR-6132 and FOXP3 play important roles in DVT.

### miR-6132 attenuates the expression of FOXP3 *in vitro*

**Figure 3:**
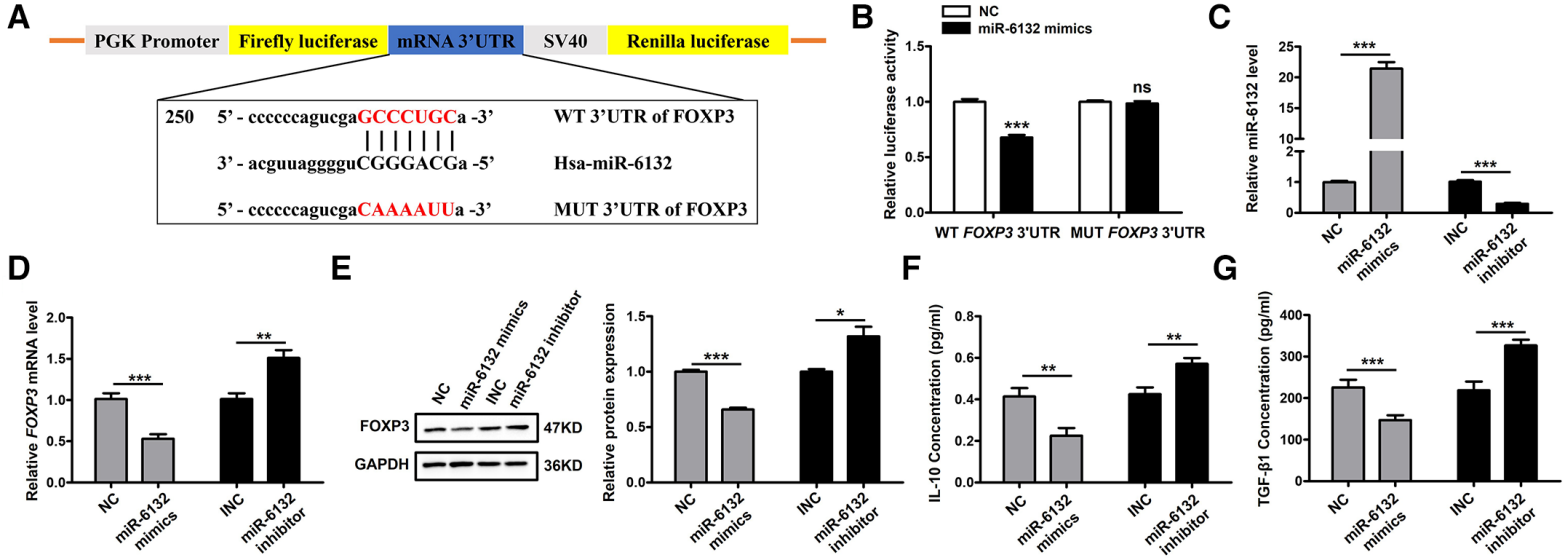
miR-6132 negatively regulates FOXP3 expression in 293T cells. (**A**) Potential miR-6132 binding sequence in *FOXP3* human mRNA 3′UTR. The sequence of the corresponding wild-type (WT) and mutant (MUT) constructs used in the luciferase reporter assay is also indicated. (**B**) The effect of negative control (NC) or miR-6132 mimics on luciferase activity transfected 293T cells expressing the WT or MUT 3′UTR of human FOXP3. (**C**) The expression level of miR-6132 after NC, miR-6132 mimics, inhibitor negative control (INC) and miR-6132 inhibitor transfection as detected by qRT-PCR. (**D**) The expression level of *FOXP3* mRNA after NC, miR-6132 mimics, INC and miR-6132 inhibitor transfection as detected by qRT-PCR. (**E**) The expression level of FOXP3 protein was detected by Western blot. (**F**,**G**) The expression levels of IL-10 and TGF-β1 protein were detected by ELISA. Results were quantified by Image J software. *P < 0.05, **P < 0.01, ***P < 0.001.

**Figure 4:**
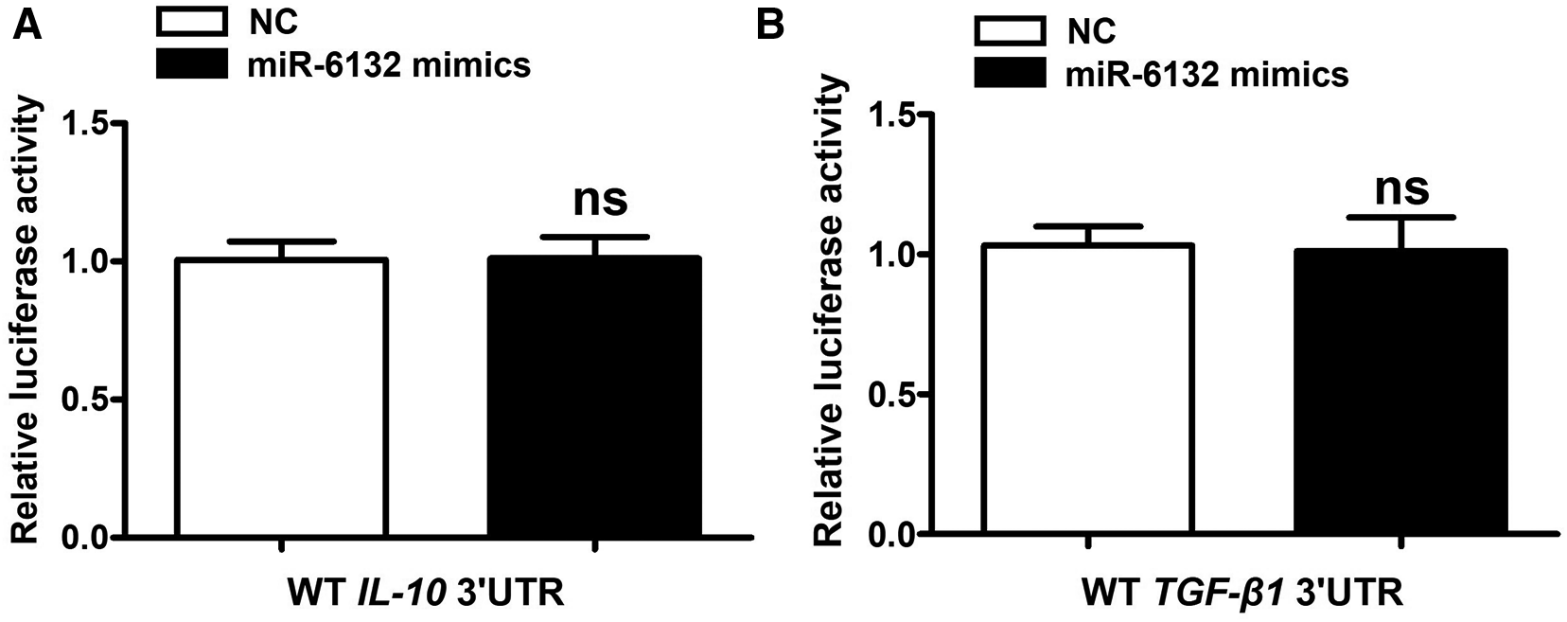
miR-6132 does not target IL-10, or TGF-β1 mRNA 3′UTR. (**A**,**B**) The luciferase activity was determined by co-transfecting the vectors (IL-10 3′UTR-WT or TGF-β1 3′UTR-WT) combined with negative control (NC) or miR-6132 mimics into 293T cells.

Database prediction discovered that miR-6132 shared binding sites with FOXP3 3′UTR (Figure 3A, Supplementary Figure S2A). Based on the dual-luciferase method, miR-6132 mimics significantly repressed luciferase activity of WT FOXP3 3′UTR, whereas did not affect MUT FOXP3 3′UTR (Figure 3B, Supplementary Figure S2B). In addition, we constructed the plasmids of WT 3′UTR and performed luciferase reporter assays to investigate whether miR-6132 might also target other factors closely related to DVT, such as IL-10 or TGF-β1. Notably, miR-6132 mimics could not inhibit the luciferase activity of IL-10 and TGF-β1 (Figures 4A,B). Taken together, our luciferase reporter analysis thus confirmed that FOXP3 is a direct target of miR-6132.

To further corroborate that FOXP3 inhibition was due to upregulation of miR-6132, we altered miR-6132 expression and determined the expression of FOXP3 in 293T cells. We observed that overexpression of miR-6132 using mimics (Figure 3C) correlated with downregulation of FOXP3 (Figures 3D,E), whereas inhibition of miR-6132 with miR-6132 inhibitor (Figure 3C) resulted in upregulation of FOXP3 (Figures 3D,E). Meanwhile, miR-6132 overexpression decreased the protein levels of IL-10 and TGF-β1 and miR-6132 knockdown promoted the protein levels of IL-10 and TGF-β1 (Figures 3F,G).

### miR-6132 inhibits the expression of FOXP3, which in turn affects the formation of DVT

**Figure 5:**
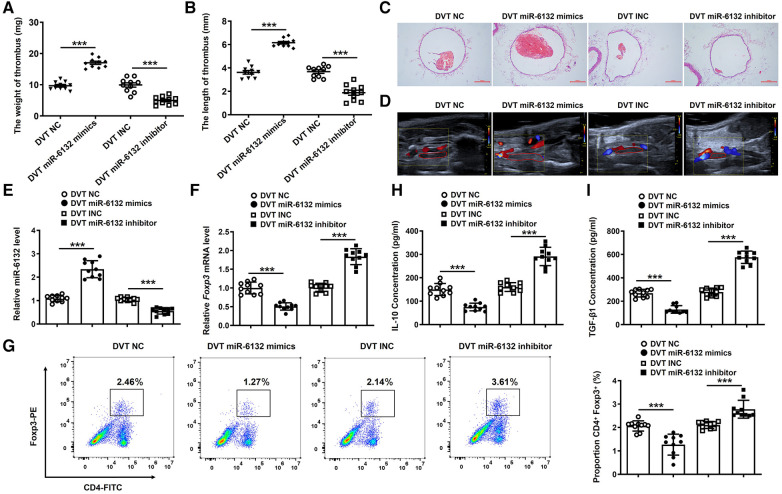
The regulatory effects of miR-6132 on the expression of FOXP3 in DVT formation. (**A**,**B**) The thrombosis weight and length in different groups (*n* = 10). (**C**,**D**) Representative images of thrombi in each group detected by H&E staining (magnification, ×100) and vascular ultrasound at 24 h post-operation. Scale bars = 200 μm. (**E**) Expression of miR-6132 was detected by qRT-PCR in spleen of each treatment group. (**F**,**G**) Foxp3 mRNA and protein levels were determined by qRT-PCR in spleen of each treatment group. (**H**,**I**) IL-10 and TGF-β1 protein levels were determined by ELISA in plasma of each treatment group. ***P < 0.001.

To extend the functional role of miR-6132 in DVT formation, miR-6132 mimics, miR-6132 inhibitor or control vectors were injected via tail vein before DVT surgery. Our results revealed that miR-6132 was significantly increased in DVT miR-6132 mimics group (Figure 5E). Similarly, H&E staining, Doppler, the thrombus length and weight showed that thrombosis was aggravated in the DVT miR-6132 mimics group at 24 h after the ligation (Figures 5A–D). We next found that Foxp3 mRNA and protein decreased in spleen of mice treated with miR-6132 mimics (Figures 5F,G), indicating that miR-6132 could negatively regulate Foxp3 expression. In contrast, miR-6132 was significantly decreased in DVT miR-6132 inhibitor group (Figure 5E), and the thrombus length and weight greatly decreased in the DVT miR-6132 inhibitor group compared with those in DVT INC group (Figures 5A–D). Meanwhile, the expression of Foxp3 increased synchronously in mice treated with miR-6132 inhibitor (Figures 5F,G).

**Figure 6:**
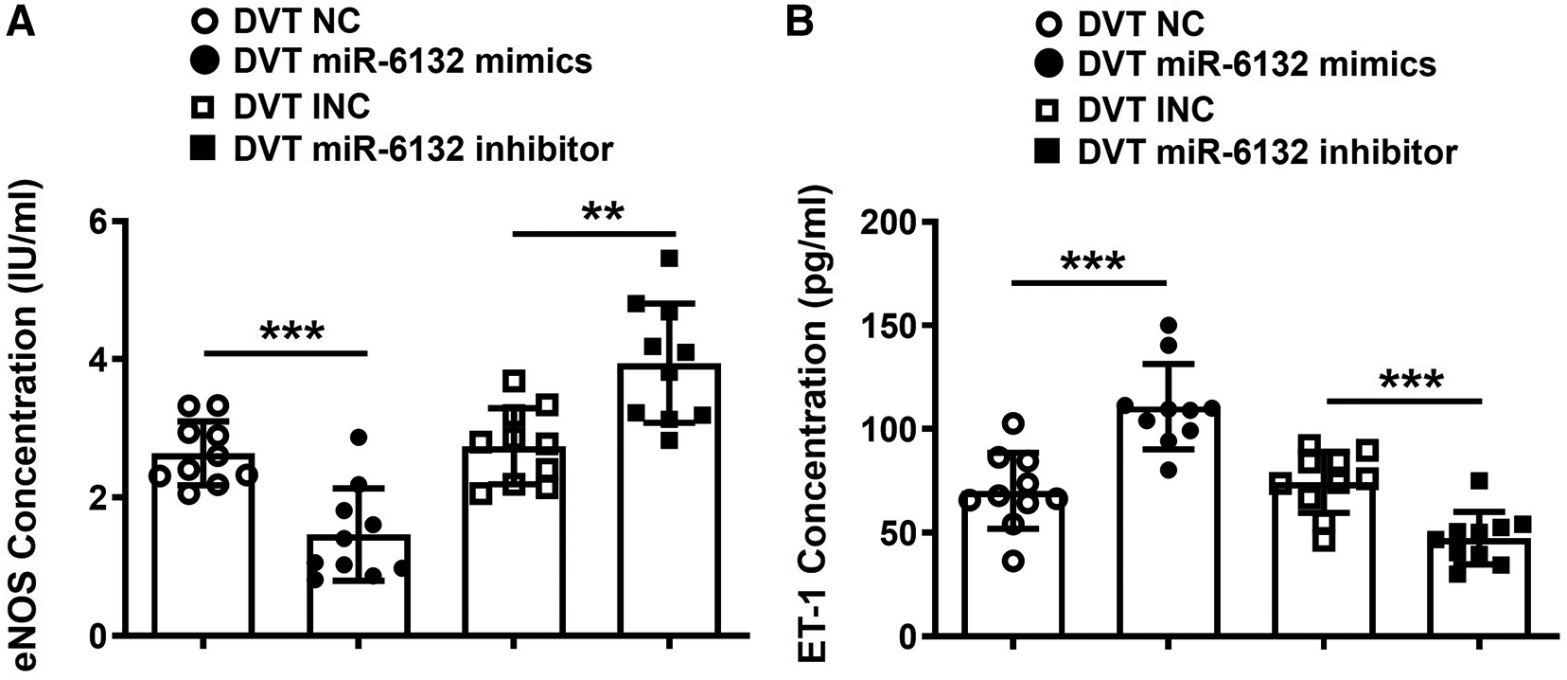
The regulatory effects of miR-6132 on the levels of eNOS and ET-1 were analyzed in plasma. (**A**,**B**) Expression of eNOS or ET-1 were determined by ELISA in plasma of each treatment group (*n* = 10). **P < 0.01, ***P < 0.001.

Moreover, expressions of IL-10, TGF-β1, and eNOS were significantly decreased, whereas ET-1 expression was increased in plasma of mice treated with miR-6132 mimics (Figures 5H,I, 6A,B). At the same time, expressions of IL-10, TGF-β1, and eNOS were obviously increased, whereas ET-1 expression was decreased in DVT miR-6132 inhibitor group (Figures 5H,I, 6A,B). These data indicated that inhibited miR-6132 could enhance FOXP3 expression and attenuate immune response, thereby alleviating endothelial dysfunction.

### Neutralization of FOXP3 promoted DVT formation *in vivo*

**Figure 7:**
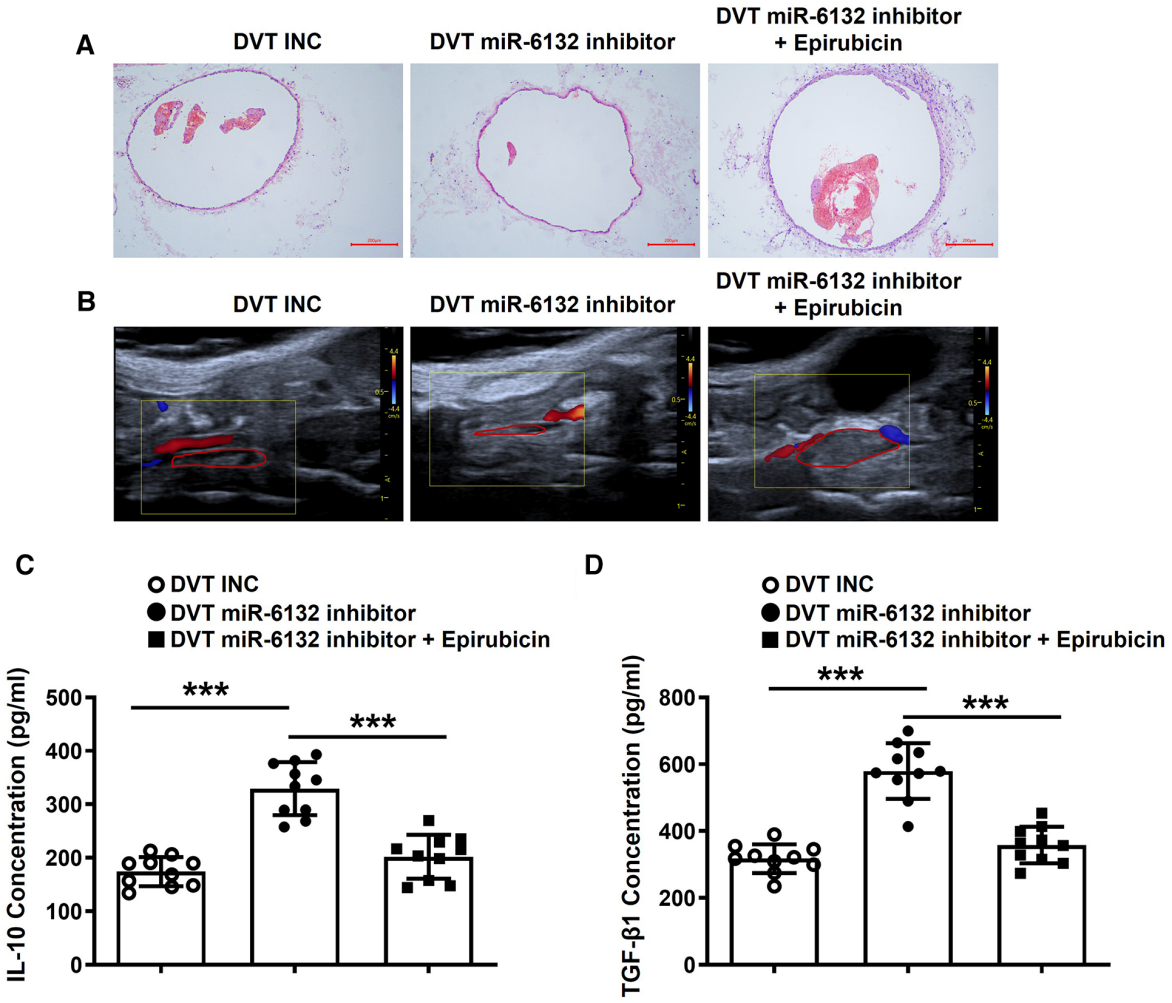
Neutralizing FOXP3 aggravated the formation of DVT *in vivo*. (**A**,**B**) Representative images of thrombi in each group detected by H&E staining and vascular ultrasound at 24 h post-operation (magnification, ×100). Scale bars = 200 μm. (**C**,**D**) IL-10 and TGF-β1 protein levels were determined by ELISA in plasma of each treatment group (*n* = 10). ***P < 0.001.

Compared with the sham groups, DVT groups showed obvious thrombosis, and IL-10 and TGF-β1 were down-regulated (Supplementary Figure S1A,B). To confirm whether Foxp3 altered the DVT formation, we injected Epirubicin into mice to neutralize Foxp3 function. Notably, the size of thrombus was significantly increased (Figures 7A,B). Analogously, IL-10 and TGF-β1 was significantly decreased after injection of Epirubicin in plasma of DVT mice (Figures 7C,D). These data indicated that FOXP3 attenuated the formation of DVT.

## Discussion

DVT is a process associated with abnormal epigenetic processes, and its pathogenesis involves complex events such as blood flow, blood components, endothelial injury, genetic predisposition, immunity, and inflammatory response (26–30). Despite increasing knowledge of risk factors, one third to one half of venous thromboembolism episodes do not have a clearly provoking cause (31, 32). The latest researches have proved that immune modulation may be involved in the occurrence and prevention of DVT (33). Here, the important role of Tregs in inducing and maintaining of immune homeostasis and tolerance, and Tregs dysfunction can lead to pathology and trigger abnormal immune responses (34). Similarly, Tregs play an indispensable role in many diseases such as autoimmune diseases, infectious diseases, organ transplantation and tumors (35–37). Evidence has demonstrated that the proportion of Tregs subgroups in DVT model rats was significantly reduced (8). Studies have reported FOXP3 has a strong regulatory effect on the function and plasticity of Treg cells, and is the main lineage master regulator for Tregs development and inhibitory activity (38–40). Since FOXP3 is controlled by transcriptional and post-transcriptional mechanisms, inhibition of FOXP3 plays an important role in tissue damage. Analogously, the study provides insights into the role of FOXP3 in DVT through epigenetic modifications.

In the past, related studies have proved the importance of miRNAs in transcriptional regulation, and some miRNAs have been widely used as biomarkers and promising cancer therapeutic targets (41, 42). The pivotal role of miRNAs has become developing new diagnostic and therapeutic tools in in cardiovascular diseases, including DVT (43–45). In our previous research, it had been identified that miR-374b-5p can promote the development of DVT, and miR-338–5p inhibited the formation of DVT (46, 23). Furthermore, the expression of FOXP3 is regulated by several miRNAs such as miR-125b, miR-133a-3p, miR-34a, and miR-210. MiR-125b and miR-133a-3p are overexpressed, thereby reducing the expression of FOXP3 and further inducing autophagy in cancer (47–49). Down-regulation of miR-34a and miR-210 induces the expression of FOXP3, a major regulator that increases Tregs function, thus alleviating allergic diseases (50, 51). Further research is necessary to explore the mechanisms that the role of miRNA regulated FOXP3 in DVT. Through TargetScan database and Chip analysis, we found miRNAs that may have regulatory effects on FOXP3. We further expanded the sample for verification, and finally found that only miR-6132 may have a targeted regulatory relationship with FOXP3.

**Figure 8:**
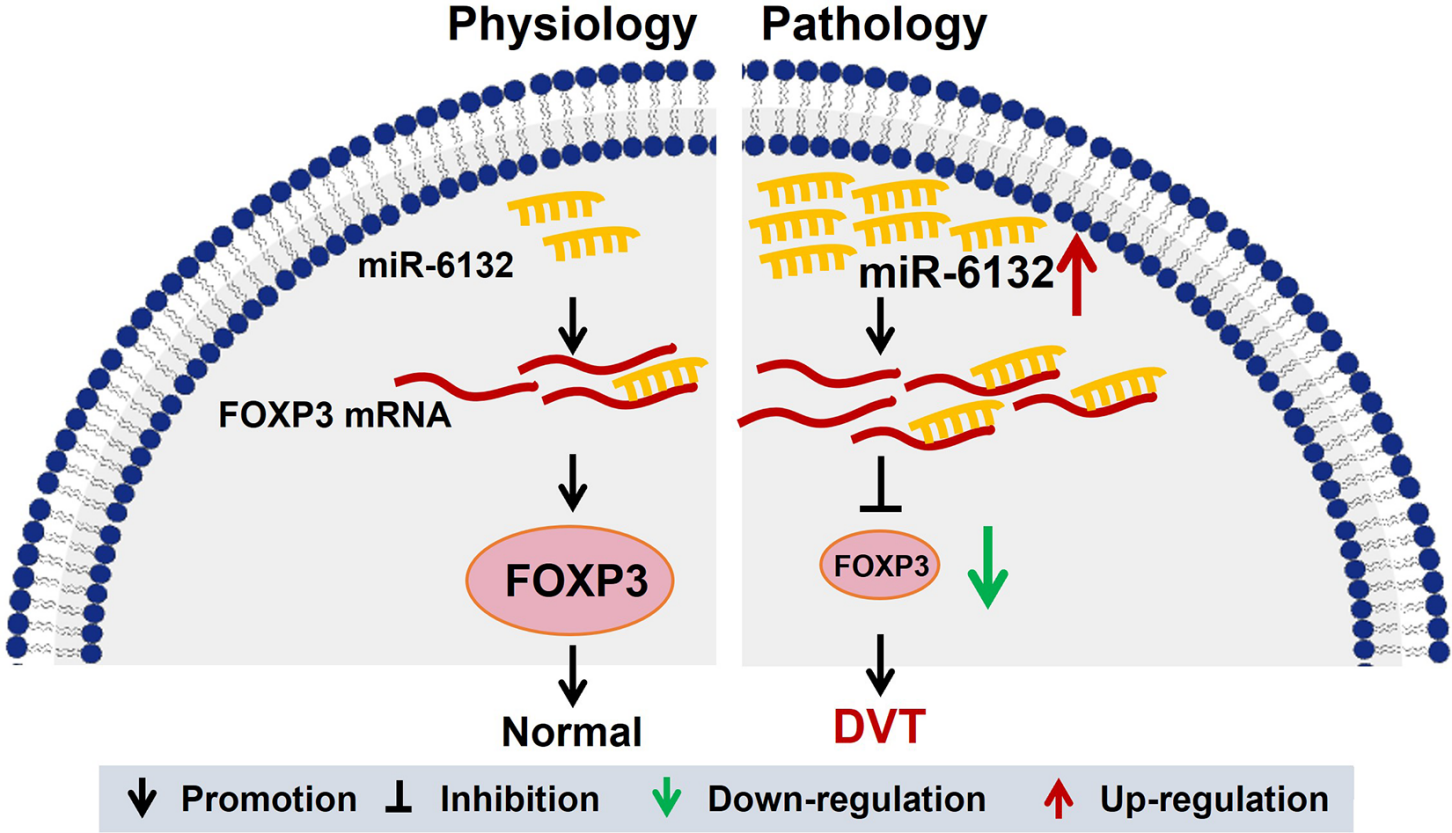
A graphical abstract showing the mechanism of miR-6132 and FOXP3 in DVT. The elevation of miR-6132 inhibited FOXP3 expression in DVT.

In many cases, microRNAs derived from the maternal gene, and the process was initiated by Drosha complex. Drosha's cleavage strictly depends on the secondary structure of the stem-loop base of pri-miRNA, as well as the sequence within the 125 nt range on the outer side of the stem-loop base. Therefore, the extremely specific nucleotides lying in the small region of sequence determined the generation of specific microRNAs. Given the large frequency of sequence variation across the species, some microRNA existed in human, but not mice, and hsa-miR-6132 is such a microRNA. To date, a mouse variant of hsa-miR-6132 has not annotated/determined. Although microRNAs are not conserved in mice, the functions of human microRNAs are usually validated in the mice model by acting on the same target gene (52, 53). In this study, we confirmed the regulatory function of hsa-miR-6132 on mouse Foxp3 with the same manner for human gene of FOXP3. To the best of our knowledge, our work provided that decreased miR-6132 increased FOXP3 expression and inhibited DVT formation (Figure 8). Thus, targeting miR-6132 may be a potentially effective strategy for DVT.

To gain a deeper understanding of the biological functions, Gene Ontology terms in Biological Process revealed that the markedly enriched pathways were the *T* cell activation signaling pathway. Here, our results showed that FOXP3 expression was obviously decreased in DVT. Coincidently, dual luciferase assay demonstrated the immediate bonding of miR-6132 to FOXP3 mRNA 3′UTR. Our pilot study showed that upregulation of miR-6132 could promote DVT formation by inhibiting FOXP3 expression. Based on the above findings, we further studied that inhibition of FOXP3 mRNA and protein levels by overexpression of miR-6132. Similarly, inhibition of miR-6132 could attenuate the formation of DVT by enhancing FOXP3 expression.

In conclusion, we first demonstrated that miR-6132 could contribute to DVT by suppressing the expression of FOXP3. The purpose of the study is to explore the biological role of miR-6132/FOXP3 axis, which will open up new potential marker for the diagnosis and treatment of DVT diseases in the future.

## Data Availability

The original contributions presented in the study are included in the article/Supplementary Materials, further inquiries can be directed to the corresponding authors.

## Supplementary material

Supplementary Figure S1 The expression of IL-10 and TGF-β1 in DVT formation. (**A**) Representative images of thrombi in each group detected by H&E staining and vascular ultrasound at 24 h post-operation (magnification, ×100). Scale bars = 200 μm. (**B**) The levels of IL-10 and TGF-β1 were detected by ELISA in plasma. ****P *< 0.001.

Supplementary Figure S2 Foxp3 was targeted and regulated by miR-6132. (**A**) Potential miR-6132 binding sequence in mouse Foxp3 mRNA 3′UTR. (**B**) The effect of negative control (NC) or miR-6132 mimics on luciferase activity expressing the wild-type (WT) and mutant (MUT) 3′UTR of mouse Foxp3. ***P *< 0.01.
