# Supplementary figures and images for: Increased miR-6132 promotes deep vein thrombosis formation by downregulating FOXP3 expression
Source: Front Cardiovasc Med. 2024 Mar 20;11:1356286. doi: 10.3389/fcvm.2024.1356286 (PMC10987872; doi:10.3389/fcvm.2024.1356286)

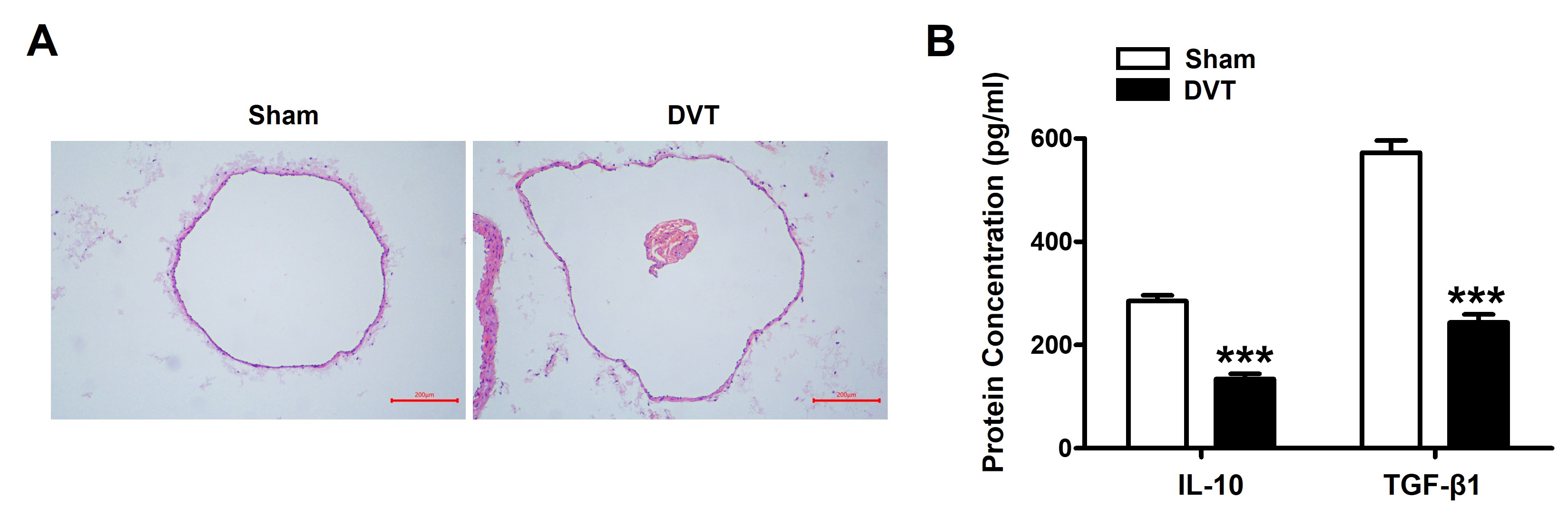

Supplement: Supplementary Figure S1 — The expression of IL-10 and TGF-β1 in DVT formation. (A) Representative images of thrombi in each group detected by H&E staining and vascular ultrasound at 24 h post-operation (magnification, ×100). Scale bars = 200 μm. (B) The levels of IL-10 and TGF-β1 were detected by ELISA in plasma. ***P < 0.001. [file Image1.jpeg]

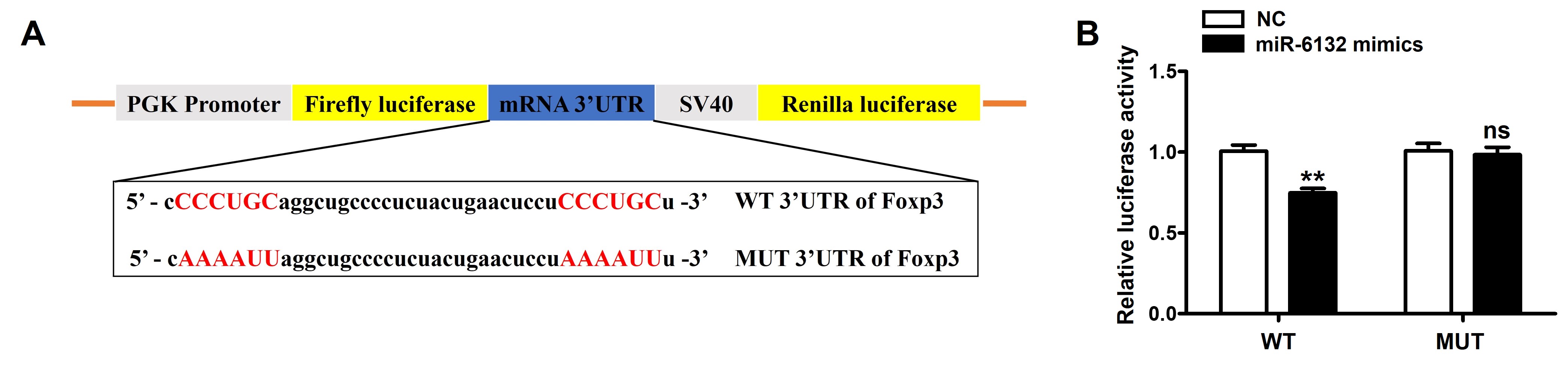

Supplement: Supplementary Figure S2 — Foxp3 was targeted and regulated by miR-6132. (A) Potential miR-6132 binding sequence in mouse Foxp3 mRNA 3′UTR. (B) The effect of negative control (NC) or miR-6132 mimics on luciferase activity expressing the wild-type (WT) and mutant (MUT) 3′UTR of mouse Foxp3. **P < 0.01. [file Image2.jpeg]
